# CpxR and LrhA coordinate the regulation of Xenocoumacin 1 biosynthesis, flagellar assembly, and chemotaxis in *Xenorhabdus nematophila*

**DOI:** 10.3389/fmicb.2026.1831682

**Published:** 2026-04-29

**Authors:** Yunfei Han, Haijiao Liu, Xintong Zhao, Mengru He, Yafei Chen, Tong Li, Juan Liu, Shujing Zhang, Gaijuan Tang, Yonghong Wang

**Affiliations:** 1 Peanut Research Institute, Henan Academy of Agricultural Sciences, Zhengzhou, Henan, China; 2 Key Laboratory of Plant Protection Resources and Pest Management, Ministry of Education, Shaanxi Biopesticide Engineering & Technology Research Center, College of Plant Protection, Northwest A&F University, Yangling, Shaanxi, China; 3 Institute of Plant Protection, Henan Academy of Agricultural Sciences, Zhengzhou, Henan, China; 4 Key Laboratory of Green Prevention and Control of Tropical Plant Diseases and Pests, Ministry of Education, School of Tropical Agriculture and Forestry (School of Agricultural and Rural Affairs, School of Rural Revitalization), Hainan University, Haikou, Hainan, China; 5 Hybrid Rapeseed Research Center of Shaanxi Province, Yangling, Shaanxi, China

**Keywords:** CpxR, LrhA, RNA-Seq, transcriptional regulation, Xenocoumacin 1 (Xcn1), *Xenorhabdus nematophila*

## Abstract

*Xenorhabdus nematophila* produces a wealth of specialized metabolites with promising agricultural and medical applications, among which Xenocoumacin 1 (Xcn1) is a key antifungal secondary metabolite. The transcriptional regulatory mechanisms governing Xcn1 biosynthesis, however, remain incompletely characterized, particularly the direct regulatory links between the two-component system response regulator CpxR and the LysR-type transcriptional regulator LrhA. Here, we combined *in vitro* protein-DNA binding assays, mutant construction, and transcriptomic analysis to dissect the coordinated regulatory roles of CpxR and LrhA in *X. nematophila* YL001. Recombinant LrhA and CpxR were heterologously expressed and purified; electrophoretic mobility shift assays demonstrated that LrhA directly binds to the promoters of *xcnA*, *lrhA*, *leuO*, and *ompR*, whereas CpxR targets the promoters of *xcnA*, *lrhA*, and *opnP.* Notably, the position of His-tag modification critically impacts LrhA’s DNA-binding activity—C-terminal tagging abrogated binding capacity, while N-terminal tagging preserved it. Deletion mutants were constructed via homologous recombination, and RNA sequencing coupled with bioinformatics analysis revealed that LrhA and CpxR exert opposing regulatory effects on overlapping core pathways: LrhA positively regulates flagellar assembly and bacterial chemotaxis, whereas CpxR negatively modulates these processes. GO and KEGG enrichment analyses further uncovered distinct regulatory roles of the two regulators in carbohydrate transport and amino acid metabolism. Collectively, our findings establish that CpxR directly activates *lrhA* transcription, and LrhA directly represses *xcnA* expression, forming a regulatory cascade that fine-tunes Xcn1 biosynthesis. This study elucidates the sophisticated transcriptional regulatory network mediated by CpxR and LrhA in *X. nematophila*, providing a theoretical basis for exploiting this bacterium and its bioactive metabolites for biotechnological applications.

## 1 Introduction

*Xenorhabdus nematophila*, a symbiont of the entomopathogenic nematode *Steinernema*, generates diverse specialized metabolites during their mutualistic symbiosis; these metabolites act synergistically to facilitate the proliferation and reproduction of both partners (Masschelein et al., 2017; Neubacher et al., 2020). Owing to the broad-spectrum biological activity of its secondary metabolites against pathogenic microorganisms (McInerney et al., 1991; Pantel et al., 2018; Zhang et al., 2024; Zhou et al., 2017) and arthropods (Incedayi et al., 2021; Mollah et al., 2020; Wei et al., 2024), *Xenorhabdus* holds substantial application potential in medicine and sustainable agriculture.

Xenocoumacin 1 (Xcn1), a key secondary metabolite of *X. nematophila* whose structure was elucidated via nuclear magnetic resonance and mass spectrometry, exhibits promising agricultural application prospects—particularly its broad antifungal activity (Han et al., 2024; McInerney et al., 1991; Neubacher et al., 2020). In the biosynthetic gene cluster of Xcn1, *xcnA-L* is responsible for Xcn1 biosynthesis, while *xcnMN* catalyzes the conversion of Xcn1 into its analogues (Masschelein et al., 2017; Park et al., 2009; Reimer et al., 2011). Seven transcriptional regulators are known to modulate Xcn1 biosynthesis including FliZ (Jubelin et al., 2013), Hfq (Bode et al., 2019) and Lrp (Engel et al., 2017) exert positive effects, whereas CpxR (Zhang et al., 2019a), LeuO (Engel et al., 2017), LrhA (Lam et al., 2024) and OmpR (Park et al., 2009) function as negative regulators. Specifically, CpxR suppresses Xcn1 production by downregulating *xcnA-L* and upregulating *xcnMN* (Zhang et al., 2019a). However, the direct regulator that binds to the *xcnA* promoter to control Xcn1 biosynthesis remains unidentified, and the regulatory relationship between CpxR and LrhA in Xcn1 biosynthesis, especially whether CpxR directly regulates LrhA via promoter binding, has not been clarified.

The production of bioactive secondary metabolites in *X. nematophila* is tightly linked to its environmental niche. The CpxA/CpxR two-component system plays a pivotal role in mediating responses to the nematode host and environmental signals: the sensor kinase CpxA perceives pH fluctuations, phosphorylates the response regulator CpxR, and thereby triggers the regulation of target genes (Zhang et al., 2025). CpxR exerts dual regulatory functions in *X. nematophila*: it positively modulates motility, virulence and lipase activity, but negatively governs protease, hemolysin and antibiotic production (Herbert Tran and Goodrich-Blair, 2009; Herbert et al., 2007; Zhang et al., 2019a). Notably, CpxR activates target operon transcription via specific promoter binding (Weatherspoon-Griffin et al., 2014). Our prior work demonstrated that CpxR represses Xcn1 production by inhibiting *xcnA-L* expression (Zhang et al., 2019a), yet whether CpxR directly binds to the *xcnA-L* promoter remains unclear. CpxR also exerts regulatory effects via either cross-talk or competitive promoter binding (Jubelin et al., 2005; Siryaporn and Goulian, 2008). Our previous study revealed that OmpR does not directly regulate CpxR (Han et al., 2025). Here, we further investigate the regulatory relationship between CpxR and other regulators like OmpR and LeuO. Additionally, CpxR enhances *X. nematophila* virulence by positively regulating *lrhA* (Herbert Tran and Goodrich-Blair, 2009), but its direct binding to the *lrhA* promoter has not been verified—an important gap given that LrhA itself is a negative regulator of Xcn1 biosynthesis.

LysR homolog A (LrhA) is a conserved LysR-type transcriptional regulator (LTTR) that directly regulates its own expression via promoter binding (Lehnen et al., 2002). It is essential for *X. nematophila* virulence against *Manduca sexta*, as well as for the control of motility, lipase activity and toxin synthesis (Richards et al., 2008), and also modulates egg hatching of its nematode symbiont by regulating bioactive small molecules (Lam et al., 2024). Notably, LrhA and CpxR exert opposing regulatory effects on multiple traits, consistent with their antagonistic roles in Xcn1 biosynthesis. In *Photorhabdus luminescens*, another nematode-symbiotic bacterium, the LrhA homolog HexA is post-transcriptionally regulated by the small RNA ArcZ, which binds to mRNA of HexA to modulate bacterial pathogenicity, symbiosis and secondary metabolite production (Engel et al., 2017; Joyce and Clarke, 2003; Neubacher et al., 2020). By contrast, the molecular mechanism underlying LrhA-mediated regulation of secondary metabolites in *X. nematophila* remains elusive, particularly regarding its DNA-binding characteristics, cascade regulation and how His-tag modification affects this binding capacity.

In this study, we purified the transcriptional regulators LrhA and CpxR and assessed their binding affinity to target promoters. We also constructed *lrhA* and *cpxR* deletion mutants of *X. nematophila* YL001 and performed systematic enrichment analysis (correlation analysis, PCA, GO and KEGG) on differentially expressed genes derived from these mutants. The aim was to clarify the direct regulatory relationship between CpxR and LrhA, and their coordinated regulation of Xcn1 biosynthesis, flagellar assembly and chemotaxis in *X. nematophila*.

## 2 Materials and methods

### 2.1 Strains and their growth conditions

*Xenorhabdus nematophila* YL001 was isolated from its symbiotic nematodes, *Steinernema* sp. YL001, which was obtained from soil samples collected in Yangling, China (Zhang et al., 2019b). The morphological and molecular characteristics of *X. nematophila* YL001 have been previously identified (Fang et al., 2008). Detailed information of strains and plasmids utilized in this study is provided in Table 1. *X. nematophila* and *E. coli* were cultured in Luria-Bertani (LB) medium at 28 °C and 37 °C, respectively. Agar and antibiotics were supplied to LB medium as needed.

**Table 1:** Bacterial strains and plasmids used in this study.

Samples		Relevant characteristics	Source
Strains	*X. nematophila* YL001	Wild-type; Amp^r^ (natural resistance)	Laboratory stock
	*X. nematophila* Δ*lrhA*	*lrhA* of *X. nematophila* YL001 was replaced by Km^r^	This study
	*X. nematophila* Δ*cpxR*	*cpxR* of *X. nematophila* YL001 was replaced by Km^r^	This study
	*E. coli* S17-1λ*pir*	Donor strain for conjugations	AngYuBio
	*E. coli* DH5α	Strain for the reproduction of recombinant pET28a	AngYuBio
	*E. coli* BL21(DE3)	Strain for protein expression	AngYuBio
Plasmids	pDM4	Suicide vector; Cm^r^, *sacB*	Laboratory stock
	pDM4-*lrhA*-Km^r^	Recombinant plasmid pDM4 for Δ*lrhA* construction	This study
	pDM4-*cpxR*-Km^r^	Recombinant plasmid pDM4 for Δ*cpxR* construction	This study
	pET28a	Source of Km^r^ gene; Vector for protein expression	Laboratory stock
	pET28a-LrhA-C_6His_	Protein expression vector of LrhA-C_6His_	This study
	pET28a-LrhA-N_6His_	Protein expression vector of LrhA-N_6His_	This study
	pET28a-CpxR-N_6His_	Protein expression vector of CpxR-N_6His_	This study

Amp^r^, Ampicillin resistance; Km^r^, Kanamycin resistance; Cm^r^, Chloramphenicol resistance.

### 2.2 DNA manipulation

Genomic DNA and plasmids were extracted separately using the Rapid Bacterial Genomic DNA Isolation Kit (Sangon, China) and the HiPure Plasmid EF Mini Kit (Magen, China), respectively, following the manufacturer’s instructions. Polymerase chain reaction (PCR) amplification was performed using Hieff Canace® Plus High-Fidelity DNA Polymerase (Yeasen, China). DNA fragments generated by PCR was purified utilizing the SanPrep Column DNA Gel Extraction Kit (Sangon, China). Recombinant plasmids were structured using the Hieff Clone® Universal One Step Cloning Kit (Yeasen, China). Primers for PCR amplification were designed with Primer Premier 5.0 software. Recombinant plasmids and mutant strains were verified by DNA sequencing (AuGCT, China).

### 2.3 Protein expression and purification

Fragments containing homologous arms for recombinant vector construction were amplified from chromosomal DNA of *X. nematophila* YL001. Primers used for heterologous expression of regulators are listed in the Table 2. The pET28a vector was digested with restriction endonuclease BamH I and Nde I (both from Takara, Japan) to generate N-terminal 6 × His-tagged expression vectors, while digestion with Nco I and Xho I (both from Takara, Japan) was conducted to construct C-terminal 6 × His-tagged expression vectors. Target fragments were cloned into the digested pET28a vector to generate recombinant plasmids pET28a-LrhA-C_6His_, pET28a-LrhA-N_6His_, and pET28a-CpxR-N_6His_. All recombinant plasmids were first transformed into *E. coli* DH5α competent cells for propagation. After verification by DNA sequencing, the correct recombinant plasmids were transformed into *E. coli* BL21 (DE3) competent cells for protein expression.

**Table 2:** Primers for protein expression.

Primers	Sequence (5′-3′)	Notes
lrhA-c6-F	TTAACTTTAAGAAGGAGATATACCATGATAAATGCAAATCGTCAGATAATA	Expression of LrhA-C_6His_
lrhA-c6-R	GCCGGATCTCAGTGGTGGTGGTGGTGGTGTTCGTCTATATTTTCGGCAGAGGT	
lrhA-n6-F	GGCCTGGTGCCGCGCGGCAGCCATATGATAAATGCAAATCGTCAGATAATA	Expression of LrhA-N_6His_
lrhA-n6-R	TGTCGACGGAGCTCGAATTCGGATCCTTATTCGTCTATATTTTCGGCAGAGGT	
cpxR-n-F	GGCCTGGTGCCGCGCGGCAGCCATATGCACAAAATCTTATTAGTTGATGATG	Expression of CpxR-N_6His_
cpxR-n-R	TGTCGACGGAGCTCGAATTCGGATCCTCATTTTACGGAAACCATTAAATATCCAC	
28a-F	GGGGTTATGCTAGTTATTGC	Validation of recombinant pET28a plasmid
28a-R	CCCAGTAGTAGGTTGAGGC	

*E. coli* BL21 (DE3) cells harboring recombinant plasmid were cultured at 37 °C with shaking at 200 rpm until the optical density at 600 nm (OD_600_) reached 1.0. Protein expression was induced by adding isopropyl-*β*-D-thiogalactopyranoside (IPTG) to a final concentration of 0.5 mM, followed by incubation with shaking overnight at 16 °C and 180 rpm. Cells were harvested by centrifugation and resuspended in lysis buffer (50 mM NaH_2_PO_4_, 300 mM NaCl, pH 8.0). Subsequently, lysozyme (final concentration 1 mg/mL), AEBSF (final concentration 2 mM), bestatin (final concentration 0.13 mM) and leupeptin (final concentration 10 μM) were added to the cell suspension, which was then incubated on ice for 30 min. Cells were lysed by ultrasonication (Scientz Biotechnology, China) with six cycles of sonication. The lysate was centrifuged at 10,000 rpm and 4 °C for 30 min to collect the supernatant, which was then applied to BeyoGold™ His-tag Purification Resin (Beyotime, China) for protein binding. Purified protein was obtained by washing the resin and eluting with a gradient of imidazole (10 ~ 100 mM imidazole, 50 mM NaH_2_PO_4_, 300 mM NaCl, pH 8.0). Eluted fractions were sampled and analyzed by sodium dodecyl sulfate-polyacrylamide gel electrophoresis (SDS-PAGE). Fractions containing LrhA-C_6His_ and LrhA-N_6His_ were pooled separately and dialyzed against phosphate buffer (10 mM Na_2_HPO_4_, 2 mM KH_2_PO_4_, pH8.0) at 4 °C. The purity of dialyzed LrhA-C_6His_ and LrhA-N_6His_ were verified by SDS-PAGE, and gel images were captured using a GelDoc XR + Gel imager (BIO-RAD, USA). Purified proteins were concentrated using a Microsep Advance Centrifugal Device (Pall Corporation, USA) and stored at −20 °C. The concentrations of purified proteins were determined using a K5800 ultra-micro spectrophotometer (KAIAO, China) prior to subsequent assays.

### 2.4 Electrophoretic mobility shift assay (EMSA)

EMSAs were performed using the aforementioned purified proteins and DNA fragments; primers for amplifying the DNA fragments are listed in Table 3. For LrhA EMSAs, 6-Carboxyfluorescein (6-FAM)-labeled DNA fragments were generated by PCR using specific primers, with one primer labeled with 6-FAM at the 5′ end. The concentrations of purified proteins and DNA probes were determined using a K5800 ultra-micro spectrophotometer (KAIAO, China). The binding reaction was prepared by mixing DNA probes, purified proteins, and 5 × EMSA reaction buffer (250 mM Tris, 250 mM KCl, 0.5 mM DTT, 50 mM MgCl_2_, 25% glycerol, pH 8.0), followed by incubation at room temperature for 30 min. The reaction was terminated by adding 5 × EMSA loading buffer (Beyotime, China), and the mixture was subsequently analyzed by 6% native polyacrylamide gel electrophoresis at 90 V in 0.5 × TBE buffer (45 mM Tris–HCl, 45 mM boric acid, pH 8.0) at 4 °C. Gel images were captured using a GenoSens transilluminator (Clinx, China). For CpxR EMSAs, gels were stained with Ultra GelRed (Vazyme, China) prior to imaging.

**Table 3:** Primers for electrophoretic mobility shift assay.

Primers	Sequence (5′-3′)	Notes
PxcnA427-F	GGTTTAAATACTCGTTTTGGTGAA	5′ 6-FAM labelled
PxcnA427-R	TTTATATTCTAATGTCAATAAAAATCACT	Promoter region of *xcnA*
PlrhA200-F	TTTGGATTGCATAAATTTTGTTAAGA	5′ 6-FAM labelled
PlrhA200-R	TGTTTATTCATCACTTTTTTTTGATT	Promoter region of *lrhA*
PleuO251-F	TTCTCTACTCCTCATTCTATCATTAT	5′ 6-FAM labelled
PleuO251-R	ACGTAGCCGTAATATTTTTATAGGCA	Promoter region of *leuO*
PcpxR156-F	GCATTAAATTTCTCCGCTTTC	5′ 6-FAM labelled
PcpxR156-R	TAATTCTTCCTCCAAGAGCAA	Promoter region of *cpxR*
PompR191-F	CCGGCCTTTGGTAATAAATCAGG	5′ 6-FAM labelled
PompR191-R	GTTTCAGCTCCCAAGATGCT	Promoter region of *ompR*
PxcnA580-F	CTCGTTTTGGTGAAGAGCAG	Promoter region of *xcnA*
PxcnA580-R	TACGTCTCGTAACGCTTCAT	
PxcnA170-F	TGAAAAGCAATTAACCCAT	Promoter region of *xcnA*
PxcnA170-R	CAATTATTTCCATACTTTCG	
PxcnA254-F	TCAATATGGAACACGACAG	Promoter region of *xcnA*
PxcnA254-R	GTTTTGGTGAAGAGCAGAC	
PopnP309-F	TGAATCAGCTCAATAAATAAGCTAAATT	Promoter region of *opnP*
PopnP309-R	TTGTTATTACCTCATTGGTGTTATTTAG	
PleuO202-F	TACGGCTACGTAAATAATAAAGCAT	Promoter region of *leuO*
PleuO202-R	TATTTCACTCCACTAAACTTATCGCA	
PlrhA203-F	TTTGGATTGCATAAATTTTGTTAAGA	Promoter region of *lrhA*
PlrhA203-R	CATTGTTTATTCATCACTTTTTTTTG	
PlrhA784-F	TTGGCAAAATGGATGATACC	Promoter region of *lrhA*
PlrhA784-R	CATTGTTTATTCATCACTTTTTTTTG	

### 2.5 Construction of mutant strains

To construct recombinant suicide vectors, the upstream and downstream homologous arm fragments of *lrhA* and *cpxR* were individually amplified from the chromosomal DNA of *X. nematophila* YL001. The kanamycin resistance gene fragment, which also contained homologous arms, was amplified separately from the pET-28a vector. Primers used for the construction and verification of mutant strains are listed in Table 4. The upstream homologous arm, downstream homologous arm, and kanamycin resistance gene fragments were ligated and cloned into the pDM4 vector, which had been digested with restriction endonucleases SphI and SacI (both from Takara, Japan). The recombinant suicide vectors were transformed into *E. coli* S17-1λpir competent cells and then conjugally transferred into *X. nematophila* YL001 (Park et al., 2009; Zhang et al., 2019a). Successful recombinant mutants were selected on LB agar plates supplemented with sucrose, ampicillin, and kanamycin.

**Table 4:** Primers for construction and verification of mutant strains.

Primers	Sequence (5′-3′)	Notes
lrhA-up-F	AGTGGGGCCCTTCTAGATAGATCTTGCATGCGAAGCCAAATGGAGCCG	5′ upstream region of *lrhA*
lrhA-up-R	ACTGAGCGTCAGTGTTTATTCATCACTTTTTTTTGATT	
lrhA-down-F	ATAGGGGTTCCGCGCTATGTGTTTTATAGGCTAA	3′ downstream region of *lrhA*
lrhA-down-R	GATAACAATTTGTGGAATCCCGGGAGAGCTCCAAACCTGCCAATAACACT	
lrhA-kr-F	GTGATGAATAAACACTGACGCTCAGTGGAACG	Kanamycin-resistant cassette
lrhA-kr-R	AAAACACATAGCGCGGAACCCCTATTTGTTT	
lrhA-in-F	CAGCGGTTAGAGCATCTT	Internal fragment of *lrhA*
lrhA-in-R	CCTTCACTTTCACCCAGA	
lrhA-out-F	AGAGTAATGTCACAATCGGTAT	External fragments of *lrhA*
lrhA-out-R	ATCAGAAATGGGAATGGA	
cpxR-up-F	AGTGGGGCCCTTCTAGATAGATCTTGCATGCATTAGGTCACGCCAGCATA	5′ upstream region of *cpxR*
cpxR-up-R	GTTTTTCTAAGAATAATTCTTCCTCCAAGAGCAAAATACGA	
cpxR-down-F	CACTGAGCGTCAGTCAATAGCCTGACAGCCCGTAT	3′ downstream region of *cpxR*
cpxR-down-R	GATAACAATTTGTGGAATCCCGGGAGAGCTCTATCCAGCGTTTTGTTCATCTGT	
cpxR-kr-F	TCAGGCTATTGACTGACGCTCAGTGGAACGA	Kanamycin-resistant cassette
cpxR-kr-R	GAGGAAGAATTATTCTTAGAAAAACTCATCGAGCATC	
cpxR-in-F	CGCAGATGACTATCTCCCT	Internal fragment of *cpxR*
cpxR-in-R	ATTAAATATCCACGACCACG	
cpxR-out-F	CTGCGGTAGTAACCAGAGT	External fragments of *cpxR*
cpxR-out-R	GGTCGGTCAAACATCAAG	
415-pDM4-F	TGGACAACAAGCCAGGGAT	Validation of recombinant pDM4 plasmid
415-pDM4-R	GCAAAGTGCGTCGGGTG	

### 2.6 RNA isolation and library preparation

Bacterial strains for RNA extraction were cultured in LB medium at 28 °C with shaking at 180 rpm until reaching the logarithmic growth phase. Total RNA was extracted using TRIzol Reagent (Invitrogen, USA). The integrity and quality of the extracted RNA were evaluated using the RNA Nano 6,000 Assay Kit on the Bioanalyzer 2,100 system (Agilent Technologies, USA). Following the isolation of mRNA from total RNA, first-strand cDNA was synthesized using random hexamer primers and M-MuLV Reverse Transcriptase. Residual RNA was then degraded by RNase H treatment. Library fragments were purified using the AMPure XP system (Beckman Coulter, USA) to select cDNA fragments of the desired size (370–420 bp). PCR amplification was performed using Phusion High-Fidelity DNA Polymerase, Universal PCR Primers, and Index (X) Primers, and the resulting products were purified using the AMPure XP system. The purified cDNA libraries were sequenced on the Illumina NovaSeq platform, generating 150 bp paired-end reads.

### 2.7 Bioinformatics analysis

Clean reads were mapped to the *X. nematophila* YL001 reference transcriptome using Bowtie 2 software (Langmead and Salzberg, 2012). The number of reads mapped to each gene was counted using HTSeq (version 0.6.1) (Anders et al., 2015). The fragments per kilobase of transcript per million mapped reads (FPKM) value for each gene was then calculated based on the gene length and the number of mapped reads. Differential expression analysis between groups was performed using the DESeq R package (version 1.18.0) (Anders and Huber, 2010). Gene Ontology (GO) enrichment analysis of differentially expressed genes (DEGs) was conducted using the GOseq R package, which corrects for gene length bias (Young et al., 2010). The statistical enrichment of DEGs in Kyoto Encyclopedia of Genes and Genomes (KEGG) pathways was analyzed using KOBAS software (Bu et al., 2021; Mao et al., 2005).

### 2.8 Reverse transcription quantitative PCR (RT-qPCR)

Total RNA was extracted using the FastPure Cell/Tissue Total RNA Isolation Kit (Vazyme, China). First-strand cDNA was synthesized using HiScript III RT SuperMix for qPCR (Vazyme, China), and qPCR amplification of target genes was performed using FastHS SYBR QPCR Mixture (Allmeek, China). RT-qPCR reactions were run on a CFX96 Touch Real-Time PCR Detection System (BIO-RAD, USA). Primers used for validating DEGs in the Δ*lrhA* and Δ*cpxR* mutants via RT-qPCR are listed in Supplementary Tables S1, S2. The relative expression levels of target genes were calculated using the 2^−ΔΔCt^ method (Pfaffl, 2001), with the housekeeping gene *recA* (Zhang et al., 2019a) serving as the internal reference to normalize the transcript levels.

## 3 Results

### 3.1 Heterologous expression of transcriptional regulators LrhA and CpxR

**Figure 1:**
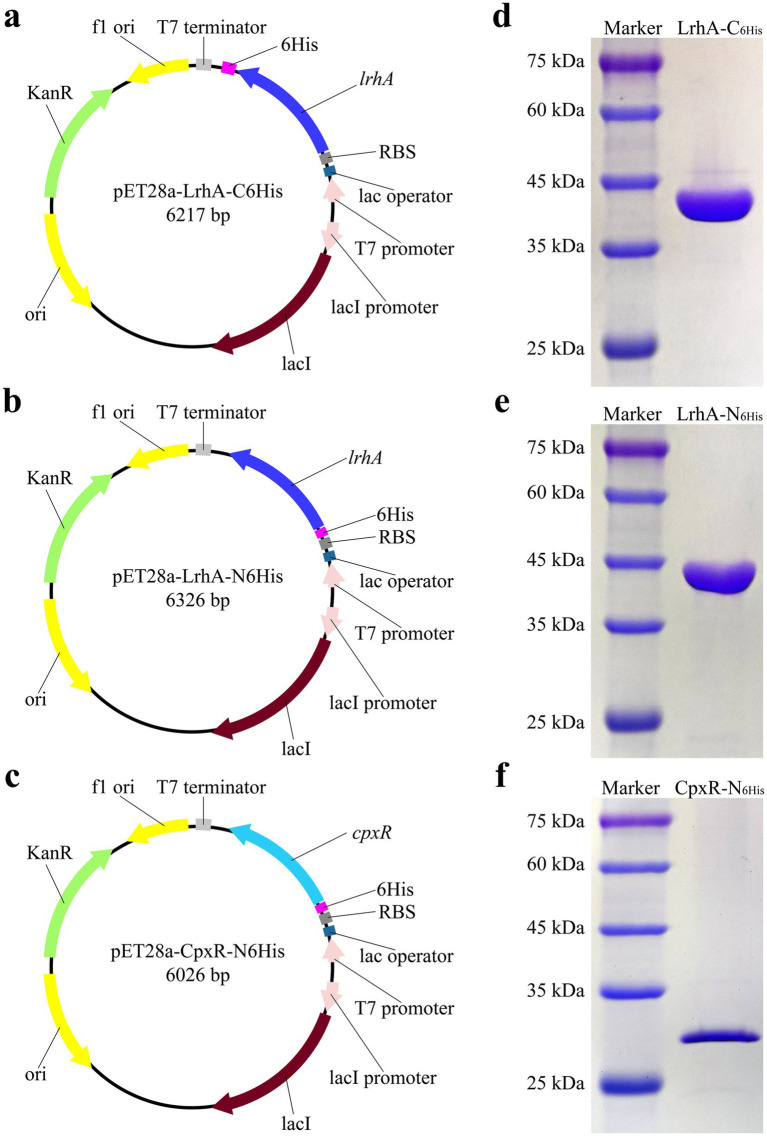
Heterologous expression of LrhA and CpxR of *X. nematophila* YL001. **(a)** Recombinant plasmid for expressing LrhA with a C-terminal 6 × His tag. **(b)** Recombinant plasmid for expressing LrhA with an N-terminal 6 × His tag. **(c)** Recombinant plasmid for expressing CpxR with an N-terminal 6 × His tag. **(d)** Purified LrhA-C_6His_ protein (37.5 kDa). **(e)** Purified LrhA-N_6His_ protein (38.8 kDa). **(f)** Purified CpxR-N_6His_ protein (28.5 kDa).

The purified DNA fragment amplified by primers with homologous arms was cloned into the plasmid pET28a digested with pairs of restriction enzyme. Recombinant vectors pET28a-LrhA-C_6His_ and pET28a-LrhA-N_6His_ were constructed, respectively, to produce LrhA labeled with 6 histidines at the carboxy and amino termini (Figures 1a,b). The recombinant vector pET28a-CpxR-N_6His_ was constructed to produce CpxR labeled with 6 histidines at the amino terminus (Figure 1c). LrhA was purified and analyzed by SDS-PAGE (Figures 1d,e). The purified LrhA-C_6His_ had a molecular weight of 37.5 kDa, and LrhA-N_6His_ was 38.8 kDa. The purified CpxR-N_6His_ was 28.5 kDa and analyzed by SDS-PAGE (Figure 1f).

### 3.2 Determination of LrhA binding to promoters of various genes

**Figure 2:**
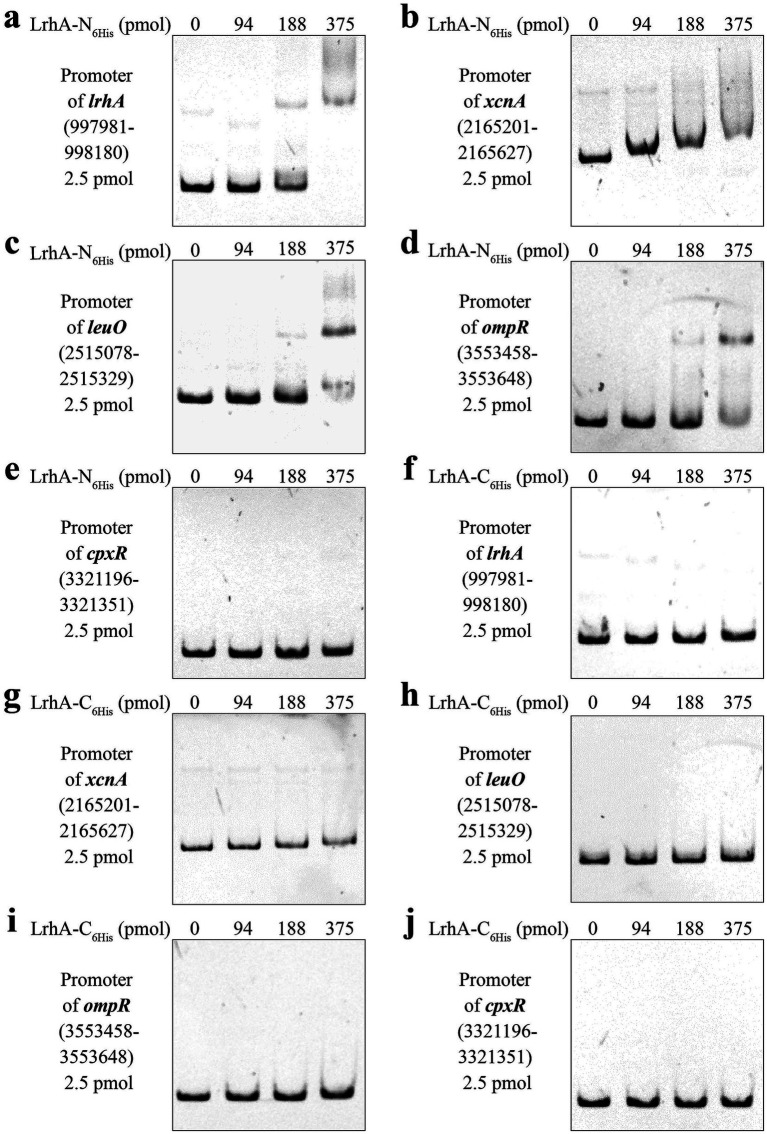
Verification of LrhA binding to the promoters of various genes. **(a)** LrhA-N_6His_ binding to the promoter of *lrhA*. **(b)** LrhA-N_6His_ binding to the promoter of *xcnA*. **(c)** LrhA-N_6His_ binding to the promoter of *leuO*. **(d)** LrhA-N_6His_ binding to the promoter of *ompR*. **(e)** LrhA-N_6His_ did not bind to the promoter of *cpxR*. **(f)** LrhA-C_6His_ did not bind to the promoter of *lrhA*. **(g)** LrhA-C_6His_ did not bind to the promoter of *xcnA*. **(h)** LrhA-C_6His_ did not bind to the promoter of *leuO*. **(i)** LrhA-C_6His_ did not bind to the promoter of *ompR*. **(j)** LrhA-C_6His_ did not bind to the promoter of *cpxR*.

The DNA-binding activity of LrhA was verified based on the observation that the retardation effect of LrhA-N_6His_ on promoter region fragments was enhanced with increasing protein concentration. As shown in Figures 2a–d, LrhA-N_6His_ exhibited specific binding to the target DNA probes. Specifically, LrhA-N_6His_ bound to the promoter regions of *lrhA* (Figure 2a), *xcnA* (Figure 2b), and *leuO* (Figure 2c). In addition, LrhA-N6His also bound to the *ompR* promoter region (Figure 2d), but showed no binding activity toward the *cpxR* promoter region (Figure 2e). In contrast, LrhA-C_6His_ failed to exhibit any detectable binding activity to these DNA probes (Figures 2f–j). Altogether, these findings demonstrate that LrhA exerts sequence-specific DNA-binding activity toward multiple target promoters related to Xcn1 biosynthesis and regulatory pathways, and that the position of His-tag modification (N-terminus vs. C-terminus) is a critical factor determining the DNA-binding capacity of LrhA.

### 3.3 Determination of CpxR binding to promoters of various genes

**Figure 3:**
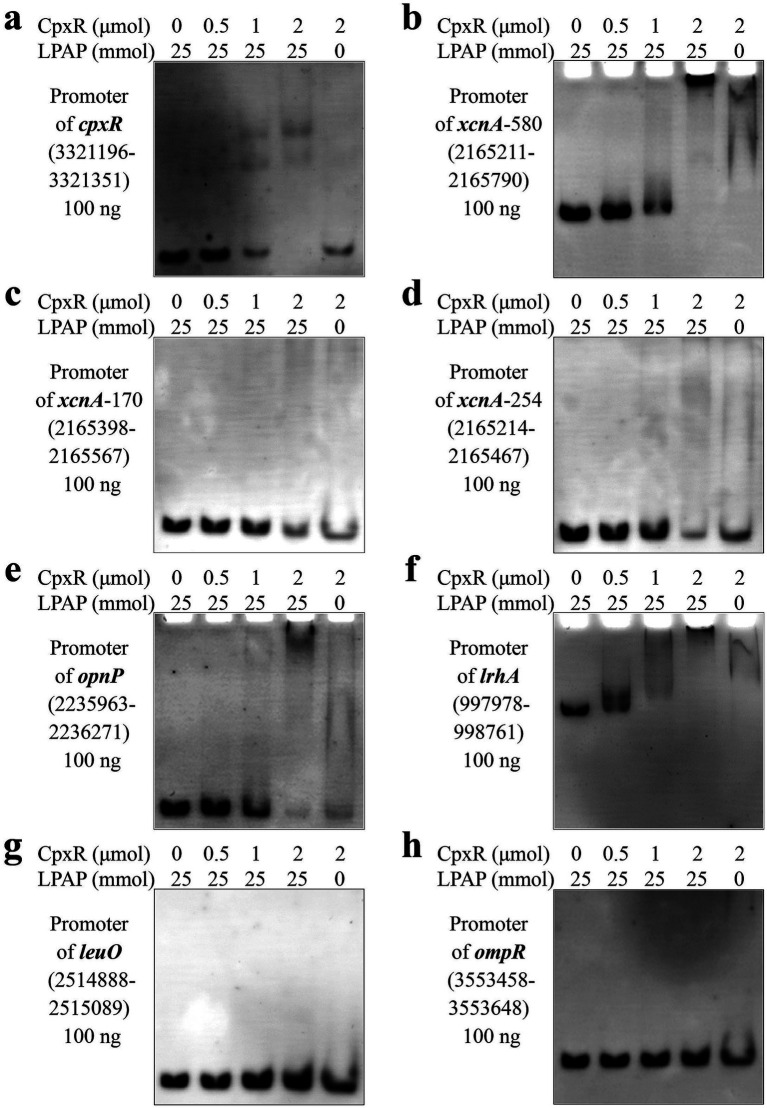
Verification of CpxR binding to the promoters of various genes. LPAP, Lithium potassium acetyl phosphate. **(a)** CpxR binding to the promoter of *cpxR*. **(b)** CpxR binding to the promoter of *xcnA* (580 bp). **(c)** CpxR binding to the promoter of *xcnA* (170 bp). **(d)** CpxR binding to the promoter of *xcnA* (254 bp). **(e)** CpxR binding to the promoter of *opnP*. **(f)** CpxR binding to the promoter of *lrhA*. **(g)** CpxR did not bind to the promoter of *leuO*. **(h)** CpxR did not bind to the promoter of *ompR*.

To validate the DNA-binding activity of the active form of CpxR in EMSA, lithium potassium acetyl phosphate (LPAP) was added to the reaction system for protein phosphorylation. The binding activity of phosphorylated CpxR was first verified using the *cpxR* promoter as a positive control, consistent with previous reports (Cao et al., 2018) (Figure 3a). Notably, CpxR bound to the *xcnA* promoter region, and the 580 bp DNA probe (Figure 3b) exhibited stronger binding affinity than the 170 bp (Figure 3c) and 254 bp probes (Figure 3d). Furthermore, we found that CpxR bound to the promoters of *opnP* (Figure 3e) and *lrhA* (Figure 3f), but did not bind to the promoters of *leuO* (Figure 3g) and *ompR* (Figure 3h). Collectively, these results demonstrate that CpxR exhibits specific binding to a subset of target promoters, including those of *xcnA* (a key Xcn1 biosynthetic gene) and *lrhA* (a negative regulator of Xcn1 biosynthesis), directly confirming the direct transcriptional regulatory links between CpxR and these critical genes in the Xcn1 biosynthetic regulatory network.

### 3.4 Quantitative analysis and differential expression profiling of genes

**Figure 4:**
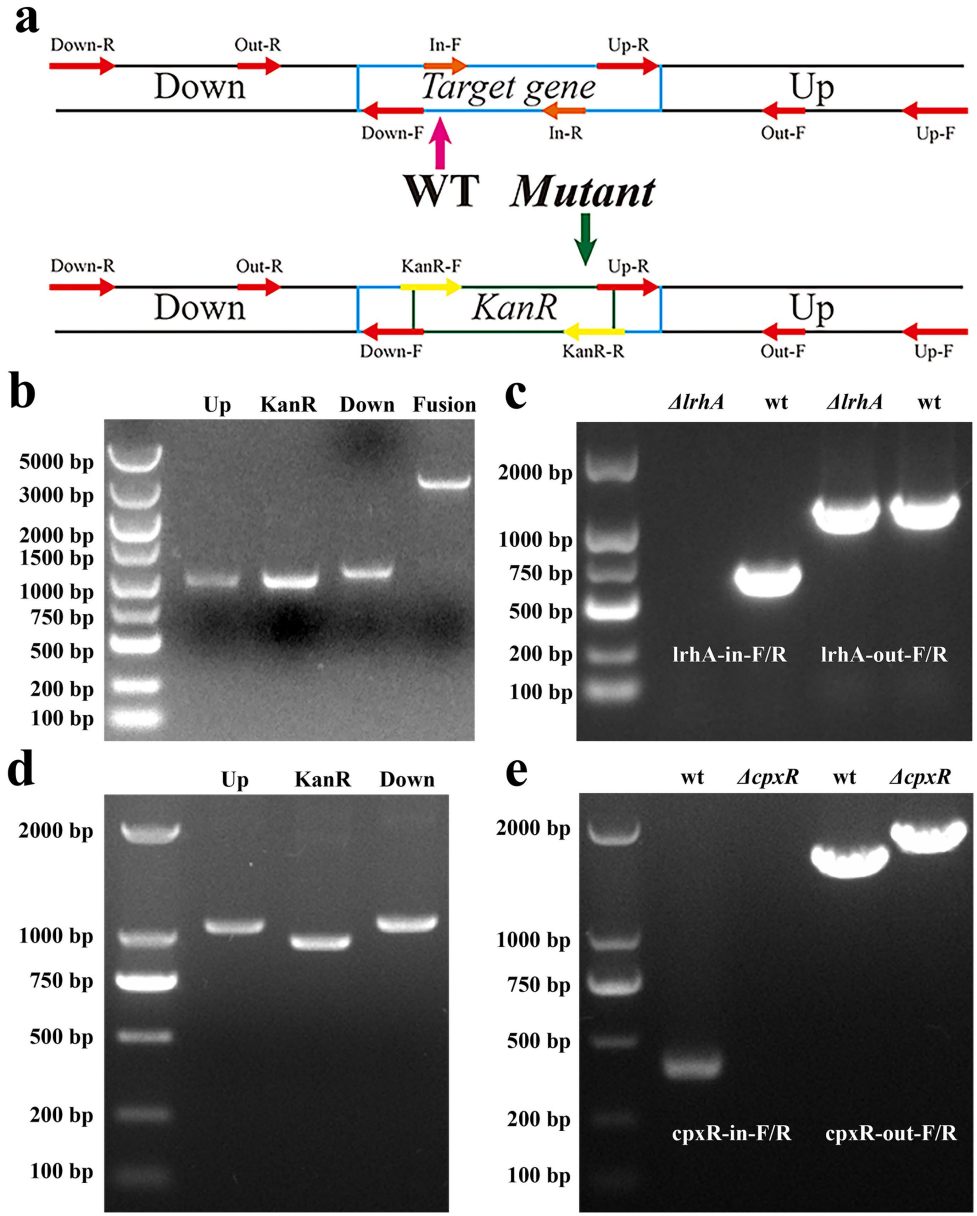
Construction and verification of Δ*lrhA* and Δ*cpxR* mutant strains. **(a)** Schematic diagram of mutant strain construction. **(b)** Amplification and fusion of target fragments for construction of Δ*lrhA*. **(c)** The identification of the Δ*lrhA* mutant using internal and external primer pairs. **(d)** Amplification of target fragments for construction of Δ*cpxR*. **(e)** Identification of the Δ*cpxR* mutant using internal and external primer pairs.

To elucidate the regulatory mechanisms of LrhA and CpxR in *X. nematophila*, RNA sequencing (RNA-seq) was performed on the wild-type (WT) strain YL001 and its derived mutants. Specifically, the *ΔlrhA* and Δ*cpxR* knockout strains were constructed via homologous recombination, in which the *lrhA* and *cpxR* coding sequences were individually replaced with a kanamycin-resistance (kanR) cassette (Figure 4). Total RNA was then extracted from each strain and subjected to high-throughput sequencing, followed by gene quantification and differential expression analysis.

**Figure 5:**
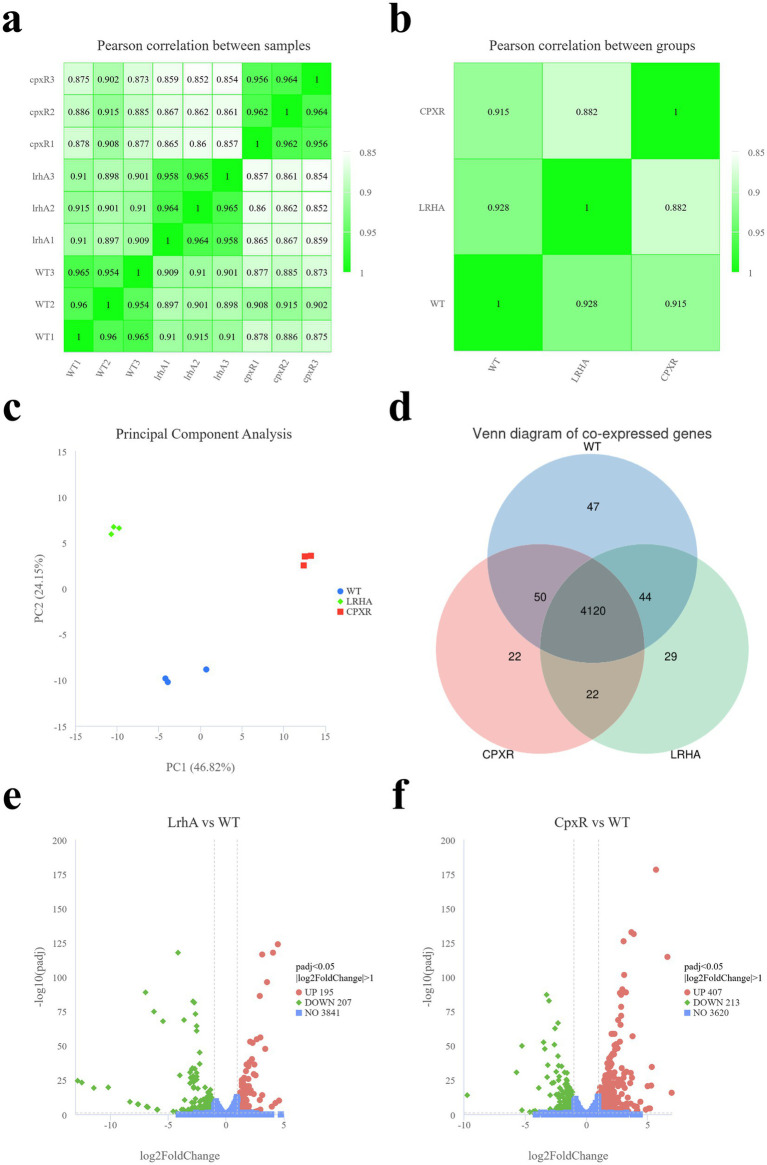
Sample correlation analysis and identification of differentially expressed genes based on RNA-Seq. **(a)** Correlation analysis of gene expression levels between samples that WT and its mutant Δ*lrhA* and Δ*cpxR*. **(b)** Correlation analysis of gene expression levels between strains that WT and its mutant Δ*lrhA* and Δ*cpxR*. **(c)** PCA of gene expression levels across all tested strains. **(d)** Venn diagram of co-expressed genes. **(e)** Volcano plot of DEGs between Δ*lrhA* and WT. **(f)** Volcano plot of DEGs between Δ*cpxR* and WT.

Pearson correlation coefficient (*R*^2^) analysis showed high correlation among biological replicates within each group, confirming the reliability of the sequencing data (Figure 5a). Intergroup correlation analysis further revealed that the transcriptomic variation in Δ*cpxR* was greater than that in Δ*lrhA* (Figure 5b). Principal component analysis (PCA) yielded a two-dimensional score plot where individual samples of the same genotype clustered tightly together; this indicated that the technical and biological variation within groups was substantially lower than the transcriptional divergence between the mutants and the WT strain (Figure 5c). In total, 4,120 co-expressed genes were detected across all tested strains (Figure 5d). Using the criteria of |log_2_ Fold Change| > 1 and adjusted *p*-value (*p*adj) < 0.05, we identified 583 upregulated and 556 downregulated genes in Δ*lrhA* relative to the WT (Figure 5e), whereas 756 upregulated and 625 downregulated genes were detected in Δ*cpxR* (Figure 5f). To validate the RNA-seq results, 20 significantly DEGs were randomly selected for RT-qPCR analysis (Supplementary Figure S1). The expression trends of these DEGs as measured by RT-qPCR were consistent with those obtained from RNA-seq, verifying the accuracy of the transcriptomic data.

### 3.5 GO enrichment analysis

To visualize the GO enrichment profiles of DEGs, a dot chart was generated using the top 10 significantly enriched terms (Supplementary Figure S2), with the significance threshold set as adjusted *p*-value (*p*adj) < 0.05. GO enrichment analysis revealed that DEGs in the Δ*lrhA* mutant were assigned to 35 distinct biological activities. Notably, downregulated genes in Δ*lrhA* were significantly enriched in 35 functional categories, which were classified into 28 biological processes (BP), 2 cellular components (CC), and 4 molecular functions (MF). The enriched BP terms included cell motility, chemotaxis, and signal transduction, et al.; the CC terms included bacterial-type flagellum and cell projection; the MF terms included secondary active transmembrane transporter activity, protein kinase activity, and phosphotransferase activity, et al. By contrast, no significant GO term enrichment was detected for the upregulated genes in Δ*lrhA*. For the Δ*cpxR*, DEGs were annotated to 41 biological activities in total. Downregulated genes in Δ*cpxR* were enriched in 1 BP term (carbohydrate transport) and 4 MF terms: transferase activity, phosphotransferase activity with an alcohol group as acceptor, and protein kinase activity, et al. Upregulated genes in Δ*cpxR* were significantly enriched in 24 BP terms, including cell motility, chemotaxis, and signal transduction, et al.; 2 CC terms (cell projection and bacterial-type flagellum); and 10 MF terms, including hydrolase activity, hydrolase activity, and transferase activity, et al. Taken together, these GO enrichment results demonstrate that LrhA and CpxR exert opposing regulatory effects on a shared set of core biological processes related to cell motility, chemotaxis, and signal transduction in *X. nematophila*—with LrhA functioning as a positive regulator and CpxR as a negative regulator of these pathways—while also mediating distinct regulatory roles in specific biological activities including carbohydrate transport and amino acid metabolism, thus expanding our understanding of the divergent yet overlapping regulatory networks governed by these two factors.

### 3.6 KEGG pathway enrichment analysis

**Figure 6:**
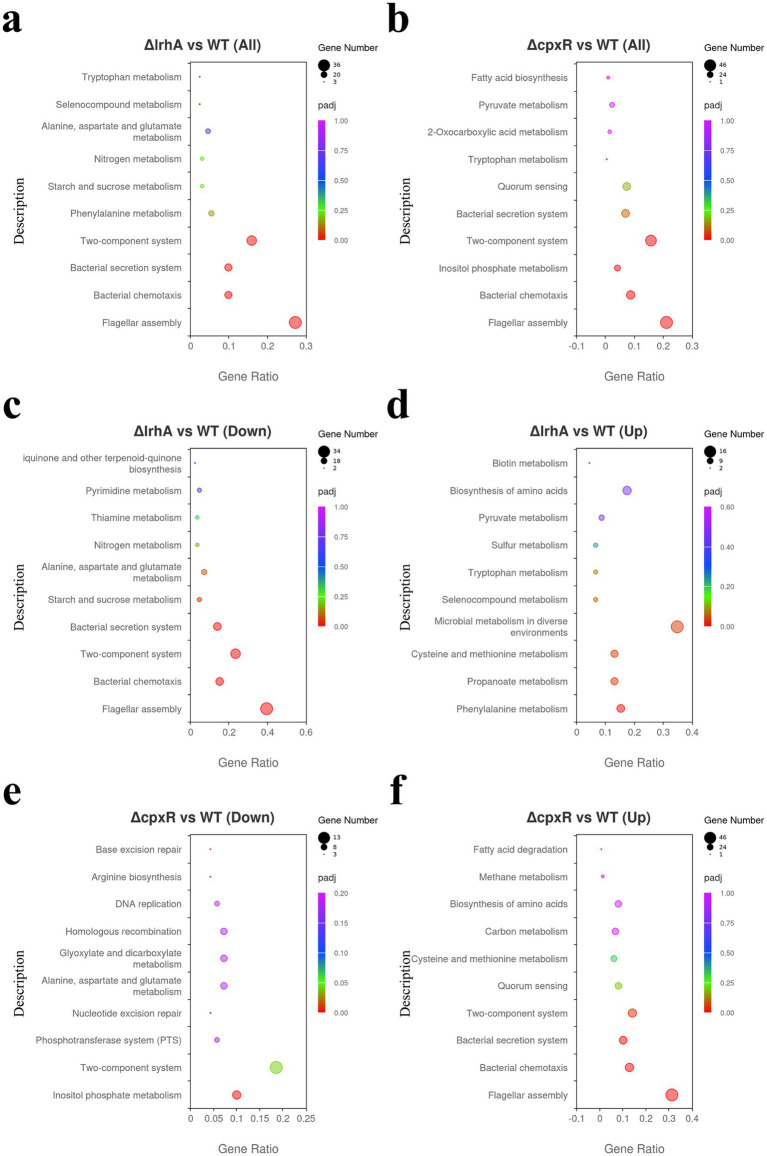
KEGG pathway enrichment analysis of differentially expressed genes. The abscissa represents the ratio of DEGs annotated to each KEGG pathway relative to the total number of DEGs, and the ordinate represents the corresponding KEGG pathway. The number of DEGs is indicated by dot size, and the dot color gradually transitions from purple to red, reflecting an increasing degree of pathway enrichment significance. **(a)** KEGG pathway enrichment analysis of DEGs between Δ*lrhA* and WT. **(b)** KEGG pathway enrichment analysis of DEGs between Δ*cpxR* and WT. **(c)** KEGG pathway enrichment analysis of downregulated genes in Δ*lrhA*. **(d)** KEGG pathway enrichment analysis of upregulated genes in Δ*lrhA*. **(e)** KEGG pathway enrichment analysis of downregulated genes in Δ*cpxR*. **(f)** KEGG pathway enrichment analysis of upregulated genes in Δ*cpxR*.

To visualize the KEGG pathway enrichment results of DEGs, a dot plot was generated using the top 10 enriched pathways. The threshold for defining significant enrichment was set as adjusted *p*-value (*p*adj) < 0.05. KEGG enrichment analysis showed that DEGs between the Δ*lrhA* and WT strain were significantly enriched in four pathways: flagellar assembly, bacterial chemotaxis, bacterial secretion system and two-component system (Figure 6a). In contrast, DEGs between the Δ*cpxR* and WT were significantly enriched in four pathways: flagellar assembly, bacterial chemotaxis, inositol phosphate metabolism, and two-component system (Figure 6b).

Further stratification by expression direction revealed that downregulated genes in Δ*lrhA* were enriched in five pathways, including flagellar assembly, bacterial chemotaxis, two-component system, bacterial secretion system, and starch and sucrose metabolism (Figure 6c), whereas upregulated genes in Δ*lrhA* were enriched in four pathways: phenylalanine metabolism, propanoate metabolism, cysteine and methionine metabolism, and microbial metabolism in diverse environments (Figure 6d). For the Δ*cpxR* mutant, downregulated genes were enriched in two pathways: inositol phosphate metabolism and two-component system (Figure 6e), while upregulated genes were specifically enriched in the flagellar assembly, bacterial chemotaxis, bacterial secretion system, and two-component system (Figure 6f). Taken together, these KEGG pathway enrichment results illustrate that LrhA and CpxR share overlapping regulatory control over the flagellar assembly, bacterial chemotaxis, and two-component system pathways in *X. nematophila*, while also exerting distinct regulatory effects on specific biological processes—such as bacterial secretion system and starch-sucrose metabolism for LrhA, and inositol phosphate metabolism for CpxR—thus revealing the conserved yet divergent roles of these two regulators in orchestrating the bacterial transcriptome and physiological functions.

## 4 Discussions

### 4.1 LrhA directly regulates the biosynthesis of active natural product

This study is the first to demonstrate that LrhA directly regulates the biosynthesis of bioactive natural products in *X. nematophila*, with a key focus on Xcn1, a major antifungal secondary metabolite of this bacterium. Specifically, our EMSA results confirmed that LrhA exerts direct transcriptional regulation on Xcn1 biosynthesis by binding to the promoter region of *xcnA*, a core gene in the Xcn1 biosynthetic gene cluster. A critical technical finding from this work relates to the impact of His-tag position on LrhA’s DNA-binding activity. As transcriptional regulators typically exert their function by directly binding to target promoters, we constructed LrhA expression vectors using the pET-28a plasmid with either N-terminal or C-terminal 6 × His tags. Published data suggested that N-terminal tags should be avoided for LTTR purification because the DNA-binding domain (DBD) is located at its N-terminus and the effector-binding domain (EBD) at its C-terminus (Baugh et al., 2023; Bundy et al., 2002). However, our experimental results revealed the opposite pattern: LrhA with a C-terminal 6 × His tag completely lost DNA-binding capacity (Figure 2), whereas N-terminal tagging preserved this function. This feature may differ from the structural characteristics of previously characterized LTTRs, for which structures are available for 16 distinct full-length LTTRs, 38 different EBDs, and 6 DBDs (Baugh et al., 2023). We compared the LTTR structures available in the UniProt^1^ database and found that LrhA exhibits substantial differences in both sequence and structure relative to other LTTRs that can be heterologously expressed using C-terminal tags. This indicates that the C-terminal region of LrhA is critical for maintaining its DNA-binding activity. Notably, LrhA-N_6His_ bound to the promoters of *lrhA* (Figure 2a) and *leuO* (Figure 2c) in *X. nematophila*, consistent with its regulatory targets in *E. coli* (Breddermann and Schnetz, 2017; Lehnen et al., 2002). This conservation in target specificity suggests functional homology of LrhA orthologs across *Enterobacterales*. Additionally, EMSA results confirmed that LrhA directly regulates *ompR*, expanding the known regulatory network of LrhA in *X. nematophila*. Further, our results reveal that LrhA exerts no regulatory effect on *cpxR*. Instead, as a direct regulator of *xcnA*, LrhA likely affects Xcn1 production by altering the expression of *leuO* and *ompR*.

### 4.2 CpxR indirectly regulates biosynthesis of Xcn1 by directly regulating LrhA

With respect to CpxR, our previous studies have shown that it negatively regulates Xcn1 production (Zhang et al., 2019a, 2019b). In this study, CpxR binds to the 580-bp promoter regionof *xcnA*, whereas no binding was detected when this promoter was truncated into 170-bp and 254-bp. Using the online software PRODORIC^2^ and FIMO,^3^ a putative CpxR-binding motif was identified on the antisense strand of the *xcnA* promoter, which accounts for the observed binding of CpxR to the 580 bp fragments. Those results indicates that Cpxr indirectly regulates the biosynthesis of Xcn1.

**Figure 7:**
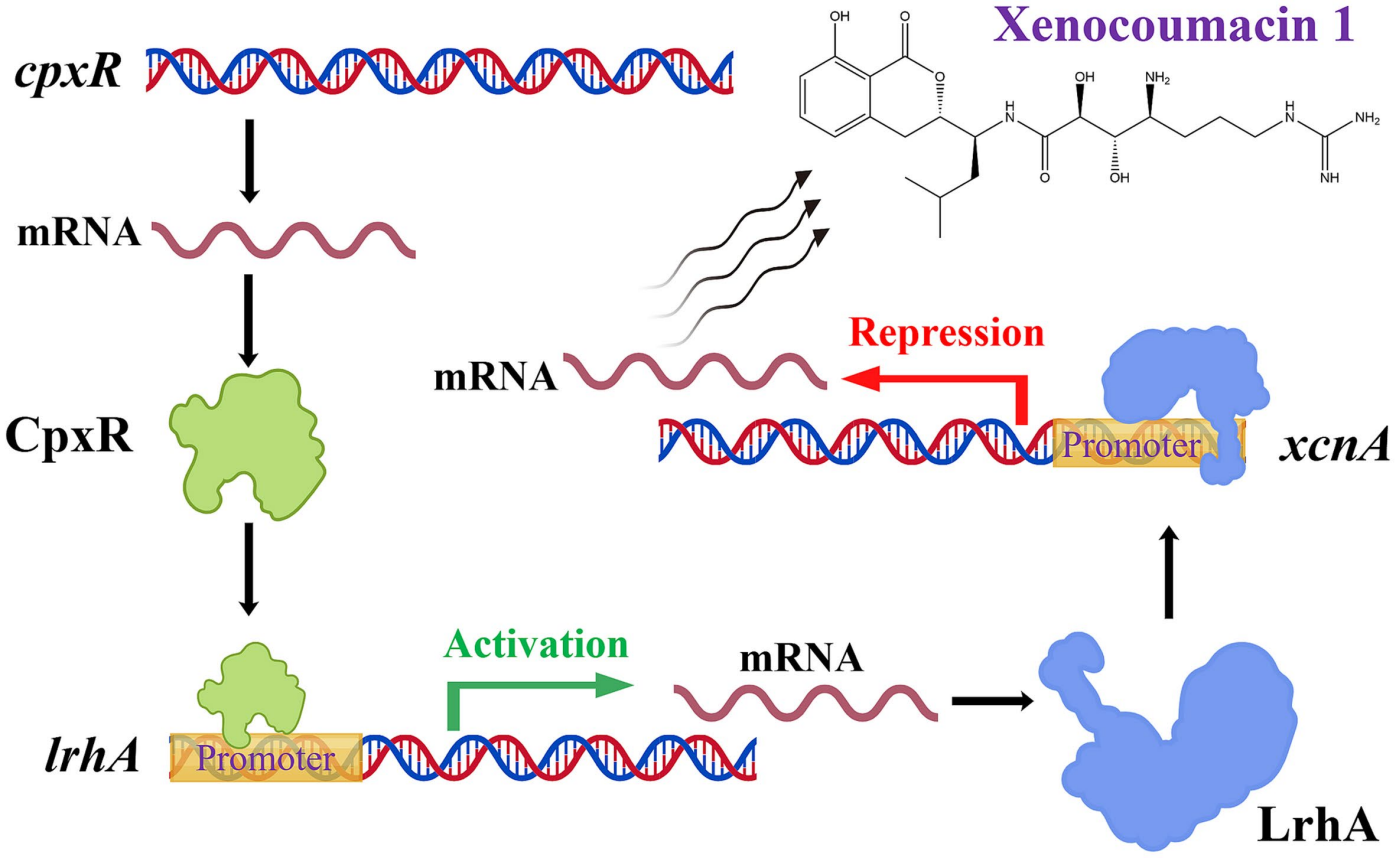
Diagram of CpxR and LrhA regulates Xcn1 biosynthesis in *Xenorhabdus nematophila*.

Furthermore, we explored the cascade regulation and competitive regulation of CpxR. CpxR binds to the promoter region of the *opnP* gene, a locus also recognized by OmpR. Both them act through binding to the same gene promoter to exert regulatory effects, in agreement with existing results (Jubelin et al., 2005). Of particular interest, our results revealed a regulatory cascade between CpxR and LrhA, while no cascade relationship existed between CpxR and OmpR or LeuO. Prior research has established that CpxR positively regulates *lrhA* (Herbert Tran and Goodrich-Blair, 2009). Current study confirmed that LrhA directly binds to the *xcnA* promoter to regulate Xcn1 biosynthesis, and that CpxR directly targets the *lrhA* promoter to control its expression. Based on the present results combined with publicly available data, we propose a cascade model governing Xcn1 biosynthesis. CpxR activates the transcription of *lrhA* via direct binding to its promoter, leading to elevated LrhA abundance. The accumulated LrhA then binds to the promoter of the *xcnA* gene and represses its expression, thereby attenuating Xcn1 biosynthesis and ultimately decreasing Xcn1 production, as shown in Figure 7 created with BioGDP.com (Jiang et al., 2025).

### 4.3 CpxR and LrhA coordinate the regulation of metabolism in *Xenorhabdus nematophila*

Compared with the WT strain, the Δ*cpxR* exhibited more extensive transcriptional differences than Δ*lrhA.* 406 and 620 DEGs were identified in Δ*lrhA* and Δ*cpxR*, respectively. GO enrichment results supposed that LrhA functions as a positive regulator, positively governing 35 biological processes. While CpxR’s regulatory functions are distributed across 41 biological processes and are primarily repressive in nature—representing a key functional distinction between the two regulators. GO enrichment analysis revealed six terms associated with motility that were negatively regulated by CpxR, including 14 flagellar structural and related genes. This finding contrasts with a previous study reporting that CpxR positively regulates bacterial motility on agar plates (Herbert et al., 2007). At present, we cannot provide a reasonable explanation for this discrepancy, and further investigation will be required in future work. KEGG enrichment analyses revealed overlapping metabolic pathways regulated by LrhA and CpxR, with opposing regulatory roles in bacterial chemotaxis, bacterial secretion system, two-component system and flagellar assembly: LrhA exerts positive regulation, while CpxR acts as a negative regulator. Additionally, existing literature has linked CpxR to bacterial virulence via LrhA regulation, and LrhA has been shown to be essential for bacterial motility, virulence, and the production of bioactive secondary metabolites (Engel et al., 2017; Herbert Tran and Goodrich-Blair, 2009; Lam et al., 2024; Richards et al., 2008). Previous studies have shown that CpxR positively regulates *lrhA* expression (Herbert Tran and Goodrich-Blair, 2009). Given our finding that LrhA and CpxR exert opposing effects on bacterial chemotaxis and flagellar assembly, speculate that CpxR and LrhA exert regulatory functions in *X. nematophila* metabolism within a more intricate regulatory network.

### 4.4 LrhA binds to gel stains, while CpxR does not

During the EMSA experiments, we observed that LrhA was stained by GelRed (Vazyme, China) and GoldView (Yeasen, China), whereas CpxR was not. This characteristic of LrhA initially interfered with our experimental process, as the gel imager could not distinguish whether the fluorescence originated from the binding of the stain to DNA or to the protein, creating ambiguity in judging the formation of DNA-protein complexes. We therefore adjusted our experimental protocol to eliminate this interference. For LrhA EMSAs, probes were labeled with 6-FAM, and gels were not stained prior to imaging. This allowed us to specifically detect the fluorescence of the labeled probes, directly reflecting the binding of LrhA to DNA. For CpxR EMSAs, probes were not labeled, and gels were stained with GelRed before imaging, consistent with conventional EMSA procedures, since CpxR does not bind to gel stains and thus does not interfere with the detection of DNA-stain fluorescence. This phenomenon is attributed to inherent structural differences between LrhA and CpxR, consistent with the functional divergences between these two regulators. Specifically, LrhA belongs to the LTTR family, which is characterized by a conserved N-terminal DBD and a variable C-terminal EBD (Baugh et al., 2023; Bundy et al., 2002); our earlier findings also revealed that LrhA’s C-terminal region is critical for maintaining its DNA-binding activity. In contrast, CpxR is a response regulator of the two-component system, possessing a typical receiver domain (for phosphorylation) and an output domain (for DNA binding) (Zhang et al., 2025). These structural distinctions likely underlie not only their differential binding to gel stains but also their distinct roles in regulating Xcn1 biosynthesis and other physiological processes.

## 5 Conclusion

This study systematically elucidates the coordinated and antagonistic regulatory mechanisms of LrhA and CpxR in *X. nematophila*, yielding three key findings: Firstly, LrhA directly regulates Xcn1 biosynthesis by binding to the *xcnA* promoter, and the position of His-tag modification (N-terminal vs. C-terminal) is critical for maintaining LrhA’s DNA-binding activity, a valuable technical insight for studies on LTTRs. Secondly, CpxR forms a regulatory module with LrhA, directly activating LrhA expression to indirectly inhibit Xcn1 biosynthesis. Finally, LrhA and CpxR regulate overlapping metabolic pathways and function oppositely in bacterial chemotaxis, secretion systems, two-component systems, and flagellar assembly, with LrhA as a positive regulator and CpxR as a negative regulator. Collectively, these findings refine our understanding of the sophisticated transcriptional regulatory networks governing secondary metabolism and motility in *X. nematophila*, providing a theoretical basis for the development of strategies to exploit *Xenorhabdus* and its bioactive metabolites in agricultural and medical applications.

## Data Availability

The original contributions presented in the study are included in the article/Supplementary material, further inquiries can be directed to the corresponding authors.

## Footnotes

1
www.uniprot.org

2
https://www.prodoric.de/matrix/

3
https://meme-suite.org/meme/tools/fimo
