# Supplementary material for: CpxR and LrhA coordinate the regulation of Xenocoumacin 1 biosynthesis, flagellar assembly, and chemotaxis in Xenorhabdus nematophila
Source: Front Microbiol. 2026 Apr 29;17:1831682. doi: 10.3389/fmicb.2026.1831682 (PMC13168091; doi:10.3389/fmicb.2026.1831682)
Supplement: Supplementary file 1 [file Supplementary_File_1.DOCX]

**CpxR and LrhA Coordinate the Regulation of Xenocoumacin 1 Biosynthesis, Flagellar Assembly, and Chemotaxis in *Xenorhabdus nematophila***

Yunfei Han^1,2,#^, Haijiao Liu^1,#^, Xintong Zhao^2^, Mengru He^2^, Yafei Chen^1,*^, Tong Li^3,*^, Shujing Zhang^4^, Gaijuan Tang^5^, Yonghong Wang^2,*^

**Supplementary Materials**

**Figure S1. RT-qPCR validation of differentially expressed genes identified by RNA-Seq**

a. Validation of RNA-Seq-identified DEGs in *ΔlrhA* by RT-qPCR. b. Validation of RNA-Seq-identified DEGs in *ΔcpxR* by RT-qPCR.


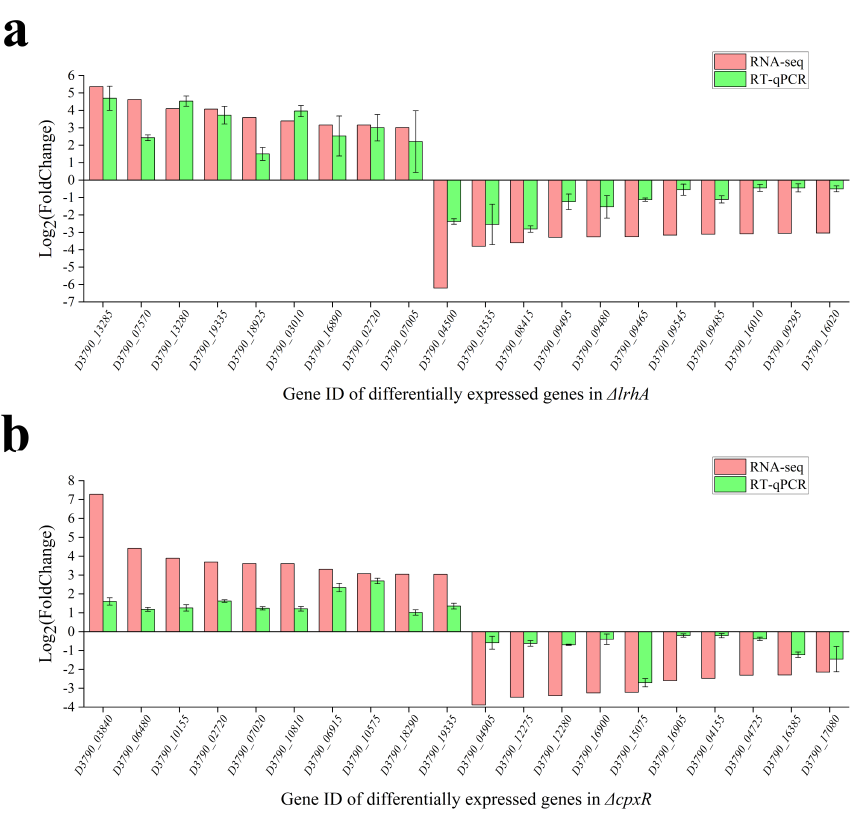


**Figure S2. GO enrichment analysis of differentially expressed genes**

The abscissa represents the ratio of DEGs annotated to each GO term relative to the total number of DEGs, and the ordinate represents the corresponding GO term. The number of DEGs is indicated by dot size, and the dot color gradually transitions from purple to red, reflecting an increasing degree of enrichment significance. a. Enriched GO terms of downregulated genes in the *ΔlrhA* compared to WT. b. Enriched GO terms of upregulated genes in the *ΔlrhA* compared to WT. c. Enriched GO terms of downregulated genes in the *ΔcpxR* compared to WT. d. Enriched GO terms of upregulated genes in the *ΔcpxR* compared to WT.


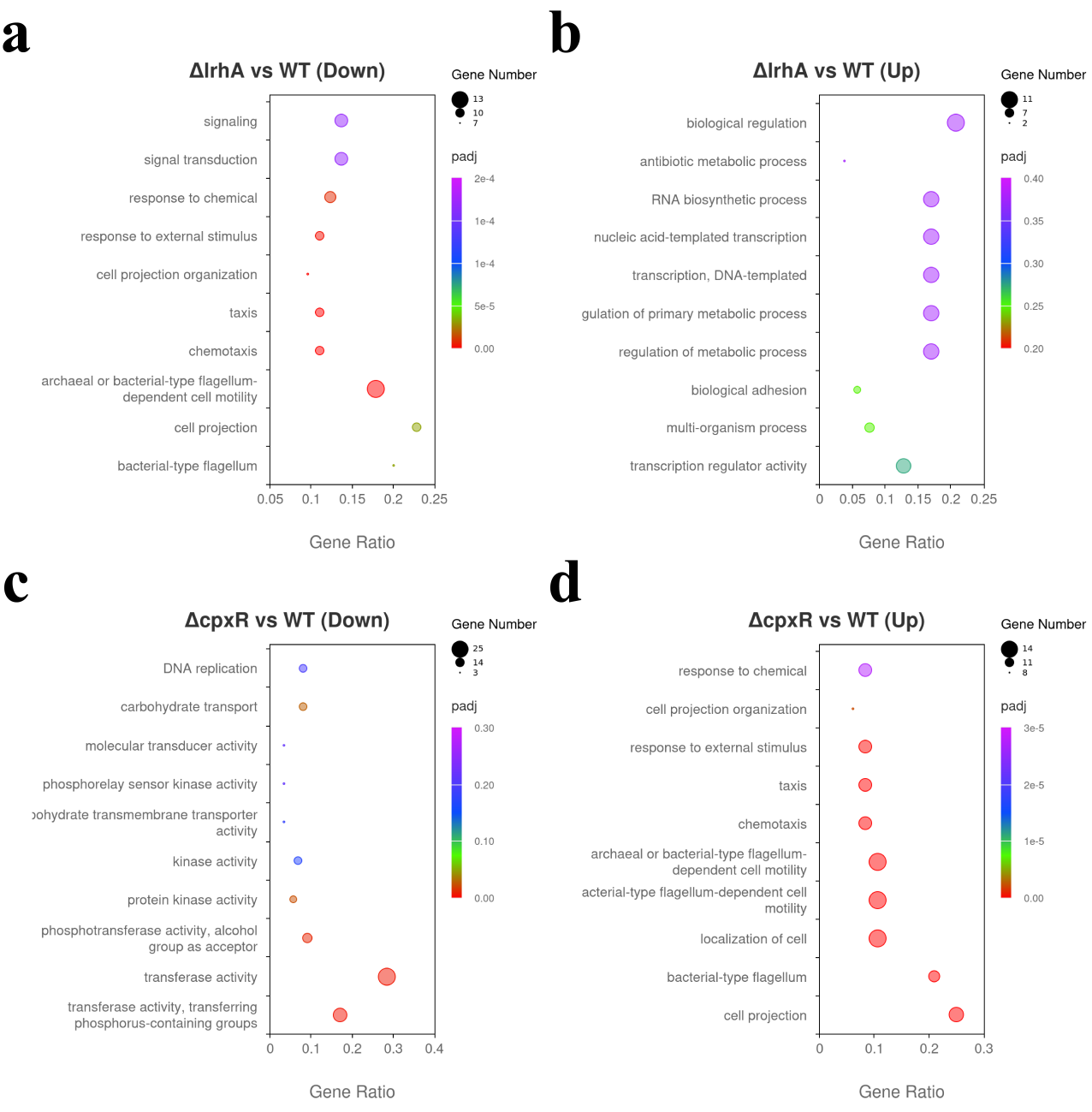


Table S1 Primers for RT-qPCR to validate differentially expressed genes in *ΔlrhA*

| Gene ID | Primers | Sequence (5'-3') | Notes |
| --- | --- | --- | --- |
| D3790_02720 | 02720-F | TCTGGGCCAGTTTCAACA | 167 bp |
|  | 02720-R | GACGCTTTCCAAATACGATA |  |
| D3790_03010 | 03010-F | CCATACCCGCTTTGAAGA | 160 bp |
|  | 03010-R | GATTTACGCCCTGCTTTC |  |
| D3790_03535 | 03535-F | GCTGAATGGCAACAACGT | 165 bp |
|  | 03535-R | CCAGTCTTCCCGCGAAAT |  |
| D3790_04500 | 04500-F | CTAGAAATGGGAATAAACTC | 174 bp |
|  | 04500-R | TTACAATAGGTGGCAGGT |  |
| D3790_07005 | 07005-F | GTGCCTGAGAAAGATGGG | 136 bp |
|  | 07005-R | TGATAGTGGGCTTGATGG |  |
| D3790_07570 | 07570-F | GGCGAAGTTTATGCCTGTC | 128 bp |
|  | 07570-R | TCACGACGCATCAGCAGT |  |
| D3790_08415 | 08415-F | TGATCGCATTTGCATCGA | 152 bp |
|  | 08415-R | CGTGTCACTTGCCGTAGA |  |
| D3790_09295 | 09295-F | GGACTTCAGCCATTCATTAGA | 195 bp |
|  | 09295-R | TGTGGATGGACACGCAAT |  |
| D3790_09465 | 09465-F | TCATCATTCAGTTGCAGGTTC | 195 bp |
|  | 09465-R | GGGCGTGGATAATCAGGT |  |
| D3790_09480 | 09480-F | TGGATGCTGCCGTTGCGGGTAT | 196 bp |
|  | 09480-R | TGCGAGCCGTGCCGATTGA |  |
| D3790_09485 | 09485-F | AAATTCTCCGGTGATAGCG | 168 bp |
|  | 09485-R | CAGGGCATGAAACTGACG |  |
| D3790_09495 | 09495-F | CCTGATAACTGCGGGAAGC | 170 bp |
|  | 09495-R | GGAGATAGGCGGTGTTCGT |  |
| D3790_09545 | 09545-F | TTCCGCACCCATTTCTTT | 159 bp |
|  | 09545-R | CGCATGATGGCTGATGTAC |  |
| D3790_13280 | 13280-F | AGCGATATTACGGGTTTG | 109 bp |
|  | 13280-R | CAGTATGGCATTCTGGTTTA |  |
| D3790_13285 | 13285-F | AAAACAACGCGGAAATAAG | 142 bp |
|  | 13285-R | TTGCTGCTACTGAAGTGAAA |  |
| D3790_16010 | 16010-F | GCTGGAAGCCATCACATT | 172 bp |
|  | 16010-R | CTCCTCGTTTCTAAGTTCTACG |  |
| D3790_16020 | 16020-F | GGGAGGTGAGAAAGTCTTCG | 225 bp |
|  | 16020-R | ATATTGACATACAGCGTGGTT |  |
| D3790_16890 | 16890-F | GAGGCCAAGGGCATAACA | 210 bp |
|  | 16890-R | GATTTCGGGCAGAAGACG |  |
| D3790_18925 | 18925-F | CTGTCTGCGTTCTCAACC | 149 bp |
|  | 18925-R | TCTTCTCCTGCGACTAAAT |  |
| D3790_19335 | 19335-F | ATGGGTCAGATAAATAACGT | 156 bp |
|  | 19335-R | TAATAAGCCTGGCATAGTG |  |
| D3790_02925 | recA-F | CAGCGTGAAGGCAGAA | 114 bp |
|  | recA-R | GGTATCAGGCTGCGAAC |  |

Table S2 Primers for RT-qPCR to validate differentially expressed genes in *ΔcpxR*

| Gene ID | Primers | Sequence (5'-3') | Notes |
| --- | --- | --- | --- |
| D3790_02720 | 02720-F | CGGCAATCTGGAGACAA | 128 bp |
|  | 02720-R | CCTGAACGAAACGCAAT |  |
| D3790_03840 | 03840-F | TCTTTAGCCAGTATTTCG | 101 bp |
|  | 03840-R | AACACCTCTTTCCCTTC |  |
| D3790_04155 | 04155-F | TTTGACCTGCCAACTCC | 102 bp |
|  | 04155-R | GTCCAGCATTCAACGAGAT |  |
| D3790_04725 | 04725-F | GCCGCTATCTTCCAACA | 100 bp |
|  | 04725-R | ACGCCATTTCCTTACCA |  |
| D3790_04905 | 04905-F | GATGTGGGATTGTCTTT | 145 bp |
|  | 04905-R | TATTGCCTATTCTGGTG |  |
| D3790_06480 | 06480-F | CTTCGGAGTTGGTTCTG | 91 bp |
|  | 06480-R | GTCATTGAAGCCGTTGG |  |
| D3790_06915 | 06915-F | AAATCAGAACAGCGTAT | 133 bp |
|  | 06915-R | AAGATGTTTCCATAGCG |  |
| D3790_07020 | 07020-F | GTATGACAACAGCCAACT | 126 bp |
|  | 07020-R | ACTCCTCCCACTATCCA |  |
| D3790_10155 | 10155-F | TACACCTGCTTCCAGTCAA | 144 bp |
|  | 10155-R | TCACGAACCCACCAATT |  |
| D3790_10575 | 10575-F | AAAATACACCTGCTTCA | 87 bp |
|  | 10575-R | TTCTTATCGCCTCAACA |  |
| D3790_10810 | 10810-F | TCGAAATACAAGGTGCT | 91bp |
|  | 10810-R | GTATCGGAAATCAGACG |  |
| D3790_12275 | 12275-F | GACAACGCATTCAGACC | 147 bp |
|  | 12275-R | TATCCGCAATGACAACA |  |
| D3790_12280 | 12280-F | CGCTGATGTAAGGAGAA | 142 bp |
|  | 12280-R | CATGGTATGCCTATTGTT |  |
| D3790_15075 | 15075-F | CTGCTGATACGGATGAT | 90 bp |
|  | 15075-R | TGCGGTAGTAACCAGAGT |  |
| D3790_16385 | 16385-F | ATCCGCATTGGTCTGTT | 111 bp |
|  | 16385-R | ACGGGTGATGGAGGGTT |  |
| D3790_16900 | 16900-F | AAGCCATCCTGAAAGCG | 143 bp |
|  | 16900-R | CATCGTGCCCAGTACATAA |  |
| D3790_16905 | 16905-F | TTAGGCGTCATAGAAAGTG | 120 bp |
|  | 16905-R | CTCTGGTTGATTGGGAAG |  |
| D3790_17080 | 17080-F | CAATCACGCAGTGGTAT | 134 bp |
|  | 17080-R | TACAGAACCCTTGGATA |  |
| D3790_18290 | 18290-F | GTATTCATCAGCACCTTTG | 83 bp |
|  | 18290-R | TATCACCCTTGCGAGTT |  |
| D3790_19335 | 19335-F | CTGAAAGATGGTGGTGAA | 131 bp |
|  | 19335-R | AATAAGCCTGGCATAGTG |  |
| D3790_02925 | recA-F | CAGCGTGAAGGCAGAAC | 114 bp |
|  | recA-R | GGTATCAGGCTGCGAAC |  |
